# Acupuncture for the treatment of allergic rhinitis: A systematic review protocol

**DOI:** 10.1097/MD.0000000000013772

**Published:** 2018-12-21

**Authors:** Haipeng Bao, Dongxu Si, Longxia Gao, Huizhuo Sun, Qi Shi, Yue Yan, Dashzeveg Damchaaperenlei, Chunlei Li, MingXia Yu, Youlin Li

**Affiliations:** a Beijing University of Chinese Medicine; b The 2nd Department of Pulmonary Disease in TCM, The Key Unit of SATCM Pneumonopathy Chronic Cough and Dyspnea, Beijing Key Laboratory of Prevention and Treatment of Allergic Diseases with TCM (No. BZ0321), Center of Respiratory Medicine, China-Japan Friendship Hospital; National Clinical Research Center for Respiratory Diseases, Beijing100029, China.

**Keywords:** acupuncture, allergic rhinitis, protocol, systematic review

## Abstract

**Background:**

Allergic rhinitis is a major chronic inflammatory disease of the respiratory tract. A large number of epidemiological investigations have shown that the prevalence of allergic rhinitis (AR) is increasing, resulting in a large burden of disease. Desensitizing drugs such as nasal glucocorticoids and antihistamines are commonly used to treat allergic rhinitis, but this method has a long treatment period and is prone to repeated attacks, and there are certain adverse reactions. Acupuncture can be used to treat a wide variety of diseases including allergic rhinitis without the occurrence of drug damage. We aim to evaluate the efficacy and safety of acupuncture in the treatment of allergic rhinitis.

**Methods:**

Relevant databases include the English databases incorporating Web of science, PubMed, Springer, Medline, Cochrane Library, EBASE, WHO International Clinical Trials Registry Platform (ICTRP), as well as the Chinese databases like the China National Knowledge Infrastructure Database (CNKI), Chinese Scientific Journal Database (VIP), Wanfang Database, and Chinese Biomedical Literature Database will be searched normatively according to the rule of each database from the inception to September 1, 2018. Reference list of identified studies, potential gray literatures, relevant conference abstracts, and clinical trial registrations will also be searched. The literature screening, data extraction, and quality assessment will be conducted by 2 researchers independently. Data will be synthesized by either the fixed-effects or random-effects model according to a heterogeneity test. Symptom score will be assessed as the primary outcome. Rhinoconjunctivitis quality of life questionnaire (RQLQ), participants with asthma can use asthma control test (ACT), medicine usage and scoring, laboratory examination, and side effects or adverse events will be evaluated as the secondary outcome. Meta-analysis will be performed using RevMan5.3.5 software provided by the Cochrane Collaboration.

**Results:**

This study will provide high-quality synthesis based on current evidence of acupuncture treatment for allergic rhinitis in several aspects, including symptom score, drug score, quality of life score, asthma control score, side effects and laboratory examination such as nasal function test, serum total immunoglobulin (IgE), nasal secretion smear, etc.

**Conclusion:**

The results of this study will provide updated evidence for weather acupuncture is an effective and safe intervention for allergic rhinitis.

**Ethics and dissemination:**

It is not necessary for this systematic review to acquire an ethical approval. This review will be disseminated in a peer-reviewed journal or conference presentation.

**PROSPERO registration number:**

PROSPERO CRD42018109105.

## 1 Introduction

Allergic rhinitis (AR) is a non-infectious inflammatory disease of the nasal mucosa, which is mainly released by immunoglobulin (IgE)-mediated mediators (mainly histamine) after contact with allergens, and involves a variety of immune-active cells and cytokines. Typical symptoms of AR are paroxysmal sneezing, watery nasal discharge, nasal itching, and nasal congestion,^[1]^ may be associated with eye symptoms, including itchy eyes, tears, red eyes, and burning sensation. It is reported that 40% of AR patients complicated with bronchial asthma, in addition to nasal symptoms, they may also have pulmonary symptoms such as wheezing, coughing, shortness of breath, and chest tightness.^[1]^ Clinical attention should be paid to the interaction and influence of AR and asthma. It is one of the most common diseases in otolaryngology, head and neck surgery (RL-HNS) and is conservatively estimated that more than 500 million AR patients worldwide,^[2]^ the prevalence of AR in the population of mainland China is 4 to 38%.^[3]^

Although rhinitis can’t be completely cured at present, through the standardized comprehensive prevention and treatment, the symptoms of patients can be well controlled and the quality of life is significantly improved. The treatment of AR includes environmental control, drug therapy, immunotherapy, and health education.^[4]^ Pollen blockers cream and cellulose powder can significantly reduce nasal symptoms and quality of life in patients with AR.^[5–7]^ Nasal glucocorticoids is currently the most effective drugs for the treatment of AR, for patients with bronchial asthma, can be conducive to asthma control and improve lung function.^[8,9]^ Other drugs, including antihistamines,^[10]^ antileukotrienes,^[11]^ mast cell membrane stabilizers,^[12]^ decongestants,^[13]^ and anticholinergics,^[14]^ are also commonly used. Allergen-specific immunotherapy has short-term and long-term effects on AR, preventing the development of asthma and reducing new sensitization.^[15]^ Some Chinese herbal medicines have anti-allergic, anti-inflammatory, and immunomodulatory effects, are effective and safe in improving the nasal symptoms of perennial and persistent AR.^[16]^

The development of acupuncture therapy has a history of thousands of years. Guided by the theory of traditional Chinese medicine, special needles are used to penetrate the patient's body according to certain acupuncture points. A certain operation method can be used to achieve the treatment through the conduction of meridians and acupoints. Acupuncture has the advantages of wide indication, obvious curative effect, convenient operation, economical and safety, etc. It can be used to treat various diseases including allergic rhinitis.^[17,18]^ Both animal experiments and clinical trials show a certain therapeutic effect of acupuncture. Studies indicated that Warm acupuncture can improve the symptoms of AR rats, which may be associated to its effect in inhibiting the expression of serum IgE, interleukin-1β (IL-1 β), and tumor necrosis factor-α (TNF-α).^[19]^ Randomized controlled trial found that acupuncture was safe and effective in the treatment of medium to severe persistent AR. Compared with the western medicine group, the acupuncture group had more advantages in the persistence of efficacy.^[20]^ Research on acupuncture for rhinitis has been increasing in recent years, and a meta-analysis on AR treated by acupuncture is necessary to conduct. In this review, we aim to systematically assess the efficacy and safety of acupuncture for AR.

## 2 Methods

### 2.1 Study registration

This systematic review protocol has been registered on PROSPERO as CRD42018109105. Available from: http://www.crd.york.ac.uk/PROSPERO/display_record.php?ID=CRD42018109105. The protocol follows the Cochrane Handbook for Systematic Reviews and Meta-Analysis Protocol (PRISMA-P) statement guidelines.^[21]^ We will describe the changes in our full review if needed.

### 2.2 Inclusion criteria for study selection

#### 2.2.1 Types of studies

In order to evaluate the efficacy and safety of acupuncture in the treatment of AR, all relevant randomized controlled trials (RCTs) published in English and Chinese on acupuncture for AR can be included. Non-RCTs, reviews, case report, experimental studies, expert experience, and duplicated publications will be excluded.

#### 2.2.2 Types of participants

Study participants in different age ranges with AR can be included in the study without restricting nationality, sex, race, occupation or education. Patients with vasomotor rhinitis, non-allergic rhinitis with eosinophilia syndrome, infectious rhinitis, hormonal rhinitis, drug-induced rhinitis, aspirin intolerance triad, cerebrospinal rhinorrhea were excluded.

#### 2.2.3 Types of interventions

The study focus on clinical trials of AR with the therapy of acupuncture, and the results will provide clinicians with consultation and advice. Thus, the experimental group treated only with acupuncture and without any combination of other drugs and treatment will be included, regardless of the number of acupoints, the method of needle insertion, duration and frequency. Combination therapy can’t evaluate the effect of acupuncture will be excluded. Studies of control groups will be treated with no treatment or sham acupuncture, placebo, and other interventions (e.g., medicine, moxibustion and other physical interventions).

#### 2.2.4 Types of outcome measures

##### 2.2.4.1 Primary outcomes

Symptom score will be assessed as the primary outcome. The main evaluation indicators of symptom score included nasal symptoms (sneezing, runny nose, nasal itching, and nasal congestion) and ocular symptoms (eye itching, foreign body sensation, red eyes, tearing). If asthma is combined, additional asthma symptoms such as wheezing, cough, shortness of breath, and chest tightness should be recorded.

##### 2.2.4.2 Secondary outcomes

The secondary outcomes of this review mainly includes the following aspects:

1. Rhinoconjunctivitis quality of life questionnaire (RQLQ), participants with asthma can use asthma control test (ACT)
2. Medicine usage and scoring
3. Nasal function examination and nasal provocation test
4. Allergen detection (e.g., skin prick test (SPT), total serum IgE detection, serum specific IgE detection will be included)
5. Supplementary examination (e.g., nasal secretion smear, determination of specific IgE in nasal lavage fluid)
6. Side effects and adverse events

### 2.3 Search methods for identification of studies

#### 2.3.1 Electronic searches

Relevant databases include the English databases incorporating Web of science, PubMed, Springer, Medline, Cochrane Library, EBASE, WHO International Clinical Trials Registry Platform (ICTRP), as well as the Chinese databases like the China National Knowledge Infrastructure Database (CNKI), Chinese Scientific Journal Database (VIP), Wanfang Database, and Chinese Biomedical Literature Database will be searched normatively according to the rule of each database from the inception to September 1, 2018.

#### 2.3.2 Searching other resources

The reference list of studies and relevant systematic reviews will be retrieved and examined for additional trials. Relevant conference abstracts will also be searched for eligible trials. In addition, we will search OpenGrey.eu as potential gray literature. ClinicalTrial.gov for ongoing and recently completed studies related to this topic will also be searched.

#### 2.3.3 Searching strategy

The search strategy for Web of science is listed in Table 1, which includes all search terms, and other searches will be conducted based on these results.

**Table 1:**
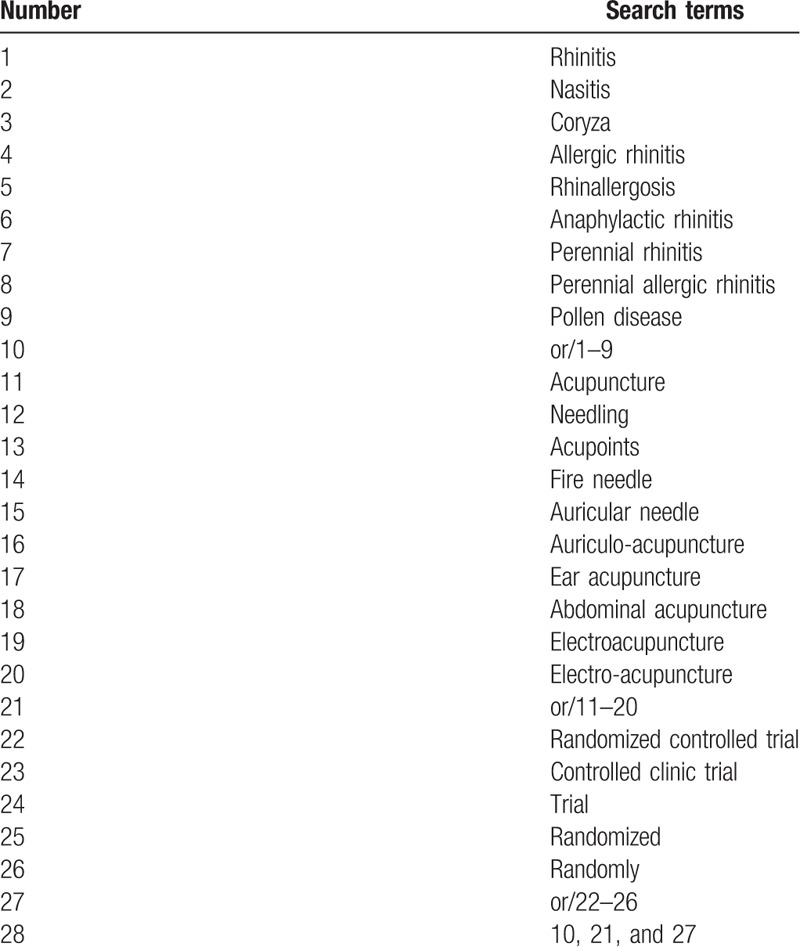
Web of science search strategy.

This search strategy will be modified as required for other electronic databases.

### 2.4 Data collection and analysis

#### 2.4.1 Selection of studies

The basic process for incorporation into the literature is determined by reference to the 5.1.0 version^[22]^ of the Cochrane Collaboration System Evaluator's Manual:

1. Obtain relevant literature by searching the specified databases, import them into the document management software Endnote X7 and screen out the duplicate documents in each database.
2. Eliminated documents that are obviously unrelated to this study by reading the titles and abstracts of the literature.
3. Download and screen the full text of the selected clinical studies related to the study
4. If there is missing or incomplete data in the study, it can be obtained through contact the study author.
5. Screening the literature included in the study according to the inclusion criteria in the literature and identified the number of papers included.

**Figure 1:**
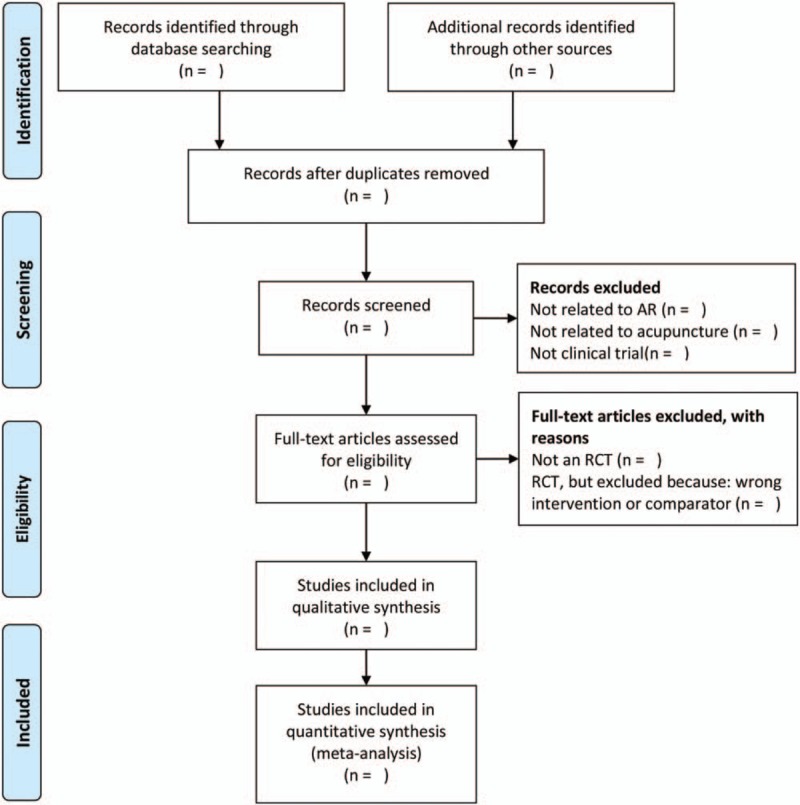
The PRISMA flow chart of the selection process. PRISMA = Systematic Reviews and Meta-Analysis Protocol.

All the procedures will be carried out by 2 independent reviewers and completed cross check. If there is any disagreement, the 3rd author will be invited to assist in the discussion and make a decision. The study of screening flow diagram is summarized in Figure 1.

#### 2.4.2 Data extraction and management

Two independent reviewers will extract data from eligible studies and enter the following information in the data extraction sheet: The basic characteristics of the study (1st author, time of publication, source/journal, and country); participant characteristics (sample size, average age, gender, inclusion and exclusion criteria, baseline situation); Interventions (type of acupuncture, randomization, allocation concealment, and blinding methods); Outcomes (measures, main outcomes, adverse effects, and follow up); If there is fund support, it will also be recorded. When a consensus on the data extraction can’t be obtained through discussion, the 3rd author will make a decision.

#### 2.4.3 Assessment of risk of bias and reporting of study quality

A rigorous quality assessment of the literature included in the study was conducted using the bias risk assessment tool of the Cochrane Collaborative Network System Evaluator’ Manual. For the methodological characteristics of the 7 items extracted in each study, High, Low, Unclear evaluations were performed for each item. Meanwhile, the STRICTA checklist will be completed.^[23]^ When a consensus on risk assessment can’t be reached by consultations, the 3rd author will make a decision.

#### 2.4.4 Measures of treatment effect

A risk ratio (RR) with 95% confidence intervals (CIs) will be used to estimate the dichotomous outcomes, and the continuous data will be analyzed by mean difference (MD) or standard MD (SMD) with 95% CIs.

#### 2.4.5 Unit of analysis issues

We will only extract the 1st experimental period data of crossover trials to avoid carryover effects. With multiple intervention groups, we will combine all relevant experimental groups and control intervention groups into a single respectively group to prevent a unit of analysis issue.

#### 2.4.6 Management with missing data

If there are missing or incomplete data, we will attempt to contact the original authors to obtain information by email or phone. If missing data are not available, the analysis will rely on available data.

#### 2.4.7 Assessment of heterogeneity

Data analysis was performed using RevMan5.3.5 software provided by the Cochrane Collaboration. Using the software to obtain forest plots and test the heterogeneity between the included studies, Chi-square test, and *I*^2^ value^[24,25]^ will be applied to calculate and present the heterogeneity degree. When *P* > .1, *I*^2^ < 50%, it is considered that there is no heterogeneity between the experiment, and the fixed effect model will be used for statistics, otherwise, the random effect model is adopt to analyze. When the number of merged articles is greater than 10, the potential publishing bias^[26]^ will be tested using an inverted funnel chart developed by Egger.^[27]^

#### 2.4.8 Assessment of reporting bias

Funnel plot will be used to assess the reporting biases when the trials included in a meta-analysis over 10.

#### 2.4.9 Data synthesis

RevMan5.3.5 will be used for all statistical analyses. Based on the heterogeneity levels of the included studies, the fixed-effects model (*I*^2^ < 50%) or random-effects model (*I*^2^ ≥50%) will be selected. The dichotomous data will be analyzed by RR with 95% CIs, while the continuous data will be analyzed by MD/SMD with 95% CIs. The meaningful heterogeneity will be explained by any additional assessment included sensitivity analysis or subgroup analysis depended on the data.

#### 2.4.10 Subgroup analysis

Subgroup analysis will be carried out according to the different types of acupuncture therapies, characteristics of participants, and outcome measures.

#### 2.4.11 Sensitivity analysis

In the case of sufficient trials data, sensitivity analysis is mainly carried out for research characteristics or types such as methodological quality, and the effects on total effect are examined by excluding certain low-quality studies or non-blinded studies.

## 3 Discussion

At present, the main treatment of AR in clinical practice is the use of antihistamines and intranasal topical glucocorticoids in the nose.^[28]^ The topical application of intranasal topical glucocorticoids is the preferred treatment for moderate and severe AR.^[29]^ Long-term use of glucocorticoids can lead to nasal dryness, epistaxis and other complications, and the incidence can reach 20%.^[4]^ In addition, the drug treatment was ineffective for some patients with moderate to severe AR.^[30]^ Although desensitization therapy has a good effect in improving symptoms, the course of disease is longer, the condition is easy to repeat and the side effects are relatively large. Therefore, non-drug interventions are urgently needed to be promoted to alleviate clinical symptoms and reduce the risk of side effects in rhinitis patients.

As a complementary and alternative medical method based on the theory of traditional Chinese medicine (TCM), acupuncture has been used in China for thousands of years. Acupuncture may modulate the immune system, it has been proposed as a useful treatment for patients with allergic rhinitis.^[30]^ Although the exact mechanism of acupuncture treatment of diseases is not utterly clear at present, results of clinical studies indicates that acupuncture therapy has a comparable effect to the medication treatment on patients with moderate to severe allergic rhinitis, and it is safe with no severe adverse effects.^[31]^ We hope this systematic review will provide more reliable evidence to help patients and clinicians in the management of allergic rhinitis.

It should be noted that there might be limitations in this review. First, the use of language including English and Chinese may induce the bias of the study. Second, different types of acupuncture, acupoints, duration, frequency, the age of patients, and degree of AR may cause high heterogeneity. Third, it is difficult to undertake single or double-blind experiment measures during acupuncture therapy.

## Author contributions

HPB, DXS and YLL contributed to conceive the idea of research, develop the search strategy, and draft the manuscript. QS and YY critically revised the manuscript and provided valuable advice on the protocol. LXG and MXY is in charge of coordination and direct implementation. HZS and DD will screen the titles, abstracts, full text and extract data independently. When HZS and DD had any disagreements about the results, CLL would act the 3rd person to coordinate. DXS and CLL will conduct statistical analysis. All review authors approved the publication of the protocol. All authors participated in the protocol design, commented on drafts of this paper, read and approved the publication of the final manuscript.

**Conceptualization:** Haipeng Bao, Dongxu Si, Youlin Li.

**Data curation:** Longxia Gao, Huizhuo Sun, Dashzeveg Damchaaperenlei, Chunlei Li.

**Formal analysis:** Dongxu Si.

**Project administration:** Haipeng Bao, Dongxu Si, Youlin Li.

**Supervision:** Qi Shi, Yue Yan, MingXia Yu.

**Writing – original draft:** Haipeng Bao, Dongxu Si.

**Writing – review & editing:** Haipeng Bao, Youlin Li.
